# Supplementary figures and images for: Lactucin, a Bitter Sesquiterpene from Cichorium intybus, Inhibits Cancer Cell Proliferation by Downregulating the MAPK and Central Carbon Metabolism Pathway
Source: Molecules. 2022 Oct 29;27(21):7358. doi: 10.3390/molecules27217358 (PMC9657596; doi:10.3390/molecules27217358)

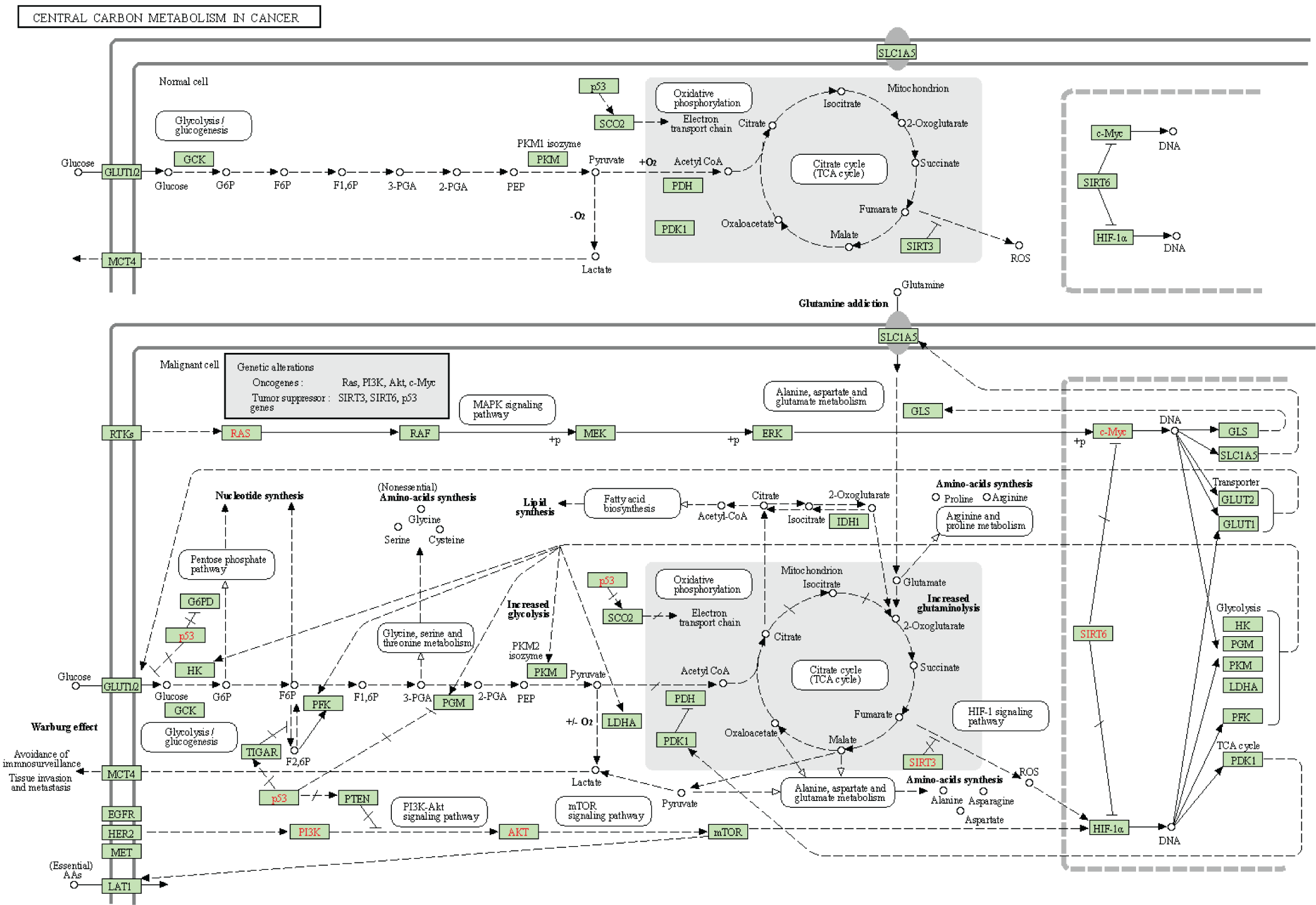

Supplement: Supplementary file 1 [file molecules-27-07358-s001.zip › Supplementary Information Figure S1.tif]

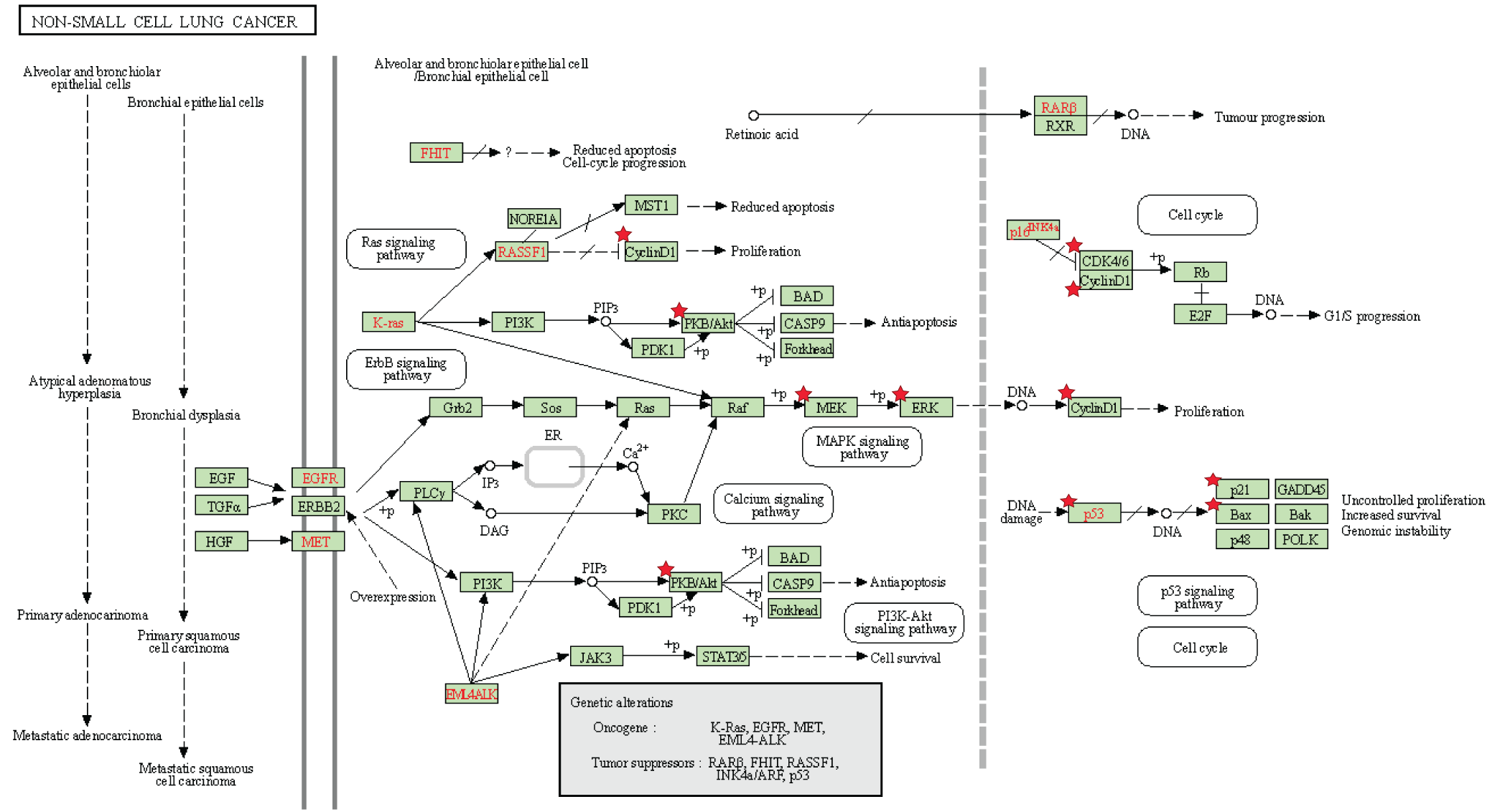

Supplement: Supplementary file 1 [file molecules-27-07358-s001.zip › Supplementary Information Figure S2.tif]
